# Review of petroleum toxicity and identifying common endpoints for future research on diluted bitumen toxicity in marine mammals

**DOI:** 10.1007/s10646-021-02373-x

**Published:** 2021-03-24

**Authors:** E. J. Ruberg, J. E. Elliott, T. D. Williams

**Affiliations:** 1 Department of Biological Sciences, Simon Fraser University, Burnaby, BC Canada; 2 Pacific Wildlife Research Centre, Environment and Climate Change Canada, Delta, BC Canada

**Keywords:** Petroleum toxicity, Marine mammals, Mini-review, Polycyclic aromatic hydrocarbons, Diluted bitumen, Oil spill

## Abstract

Large volumes of conventional crude oil continue to be shipped by sea from production to consumption areas across the globe. In addition, unconventional petroleum products also transverse pelagic habitats; for example, diluted bitumen from Canada’s oilsands which is shipped along the Pacific coast to the United States and Asia. Therefore, there is a continuing need to assess the toxicological consequences of chronic and catastrophic petroleum spillage on marine wildlife. Peer-reviewed literature on the toxicity of unconventional petroleum such as diluted bitumen exists for teleost fish, but not for fauna such as marine mammals. In order to inform research needs for unconventional petroleum toxicity we conducted a comprehensive literature review of conventional petroleum toxicity on marine mammals. The common endpoints observed in conventional crude oil exposures and oil spills include hematological injury, modulation of immune function and organ weight, genotoxicity, eye irritation, neurotoxicity, lung disease, adrenal dysfunction, metabolic and clinical abnormalities related to oiling of the pelage, behavioural impacts, decreased reproductive success, mortality, and population-level declines. Based on our findings and the body of literature we accessed, our recommendations for future research include: 1) improved baseline data on PAH and metals exposure in marine mammals, 2) improved pre- and post-spill data on marine mammal populations, 3) the use of surrogate mammalian models for petroleum toxicity testing, and 4) the need for empirical data on the toxicity of unconventional petroleum to marine mammals.

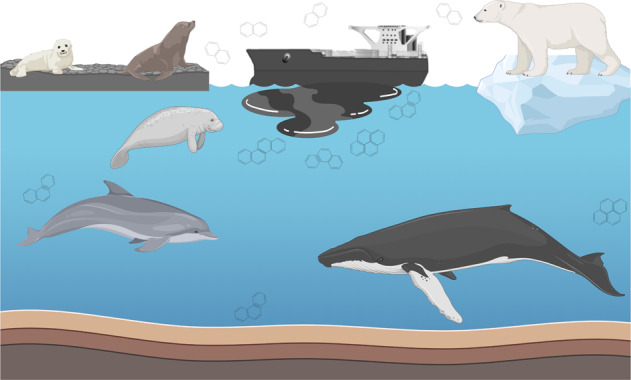

## Introduction

Conventional crude oil is a complex mixture of thousands of organic compounds, primarily hydrocarbons, along with trace elements (Kennedy 2015; Ylitalo et al. 2017). Of those, the aliphatic hydrocarbons, naphthenic acids, monoaromatic hydrocarbons, and polycyclic aromatic hydrocarbons (PAHs) are considered contaminants of toxicological concern (Kennedy 2015). As PAHs are one of the most toxic components of crude oil, they are commonly used as a predictor of toxicity (Kennedy 2015). Low molecular weight PAHs partition to and tend to remain in water, whereby they may be ingested or respired by marine mammals (Marsili et al. 2001). Consequently, those are considered to be more toxic to marine fauna than high molecular weight PAHs (Neff 1979) which partition to both organic and inorganic particles in the water column (Marsili et al. 2001). Moreover, the solubility of PAHs will increase with temperature, becoming more bioavailable for uptake by marine organisms during hotter months (Marsili et al. 2001). Global fossil fuel use and associated spillages have caused concern regarding the impacts of petroleum hydrocarbons to marine fauna (Neff 1990; Ylitalo et al. 2017). While the majority of petroleum released into the environment is through infrequent but catastrophic spills, small spills are more common and prevalent globally (Anderson and LaBelle 1994) but the effects of these small chronic spills are rarely studied (Murphy et al. 2016).

While little is known regarding the toxicity of unconventional crude oils such as diluted bitumen (dilbit) some effects can be inferred as dilbit spills have occurred, for example, the Kalamazoo River dilbit spill in 2010 (Murphy et al. 2016). Dilbit has many similar properties to conventional crude oil (Dew et al. 2015; Madison et al. 2015; Philibert et al. 2016). Although the aromatic fraction of both bitumen and conventional heavy crude oil is roughly 25–32% (Woods et al. 2008) the type and concentration of aromatic compounds will vary. For example, of the 16 US EPA Priority PAHs known to exert toxicity, when compared to samples of light, medium, and heavy conventional crude oils, dilbit contains a greater concentration of chrysene, fluorene, pyrene, fluoranthene, benzo[b]fluoranthene, benzo[k]fluoranthene, benzo[a]pyrene, dibenzo[ah]anthracene, and benzo[ghi]perylene (National Academies of Sciences, Engineering, and Medicine 2016). Consequently the PAH content of dilbit alone could predict toxicity to wildlife. In addition, bitumen contains approximately 10% more saturates, 6.5% more asphaltenes, and 6% more resins than conventional heavy crude oil (Woods et al. 2008; Dew et al. 2015). Of note, the toxic heavy metals vanadium and nickel are present in those saturate, asphaltene, and resin fractions (National Academies of Sciences, Engineering, and Medicine 2016). Finally, naphthenic acids are present in bitumen and are of concern due to their cytotoxicity and potential to disrupt the endocrine system (Headley and McMartin 2004). Because dilbit is a complex mixture of chemicals of which some are known to be toxic, the potential for additive, synergistic, or antagonistic toxicity exists. In particular, studies in fish have demonstrated that mixtures of PAHs and metals elicit more-than-additive toxicity (Gauthier et al. 2014).

If spilled into a water body, transport of dilbit initially will be similar to other commonly transported conventional crude oils (National Academies of Sciences, Engineering, and Medicine 2016). Similar to conventional crude, it is expected that the toxic volatiles benzene, toluene, ethyl benzene and xylenes (BTEX) in diluted bitumen, would quickly evaporate. Small cetaceans for example, could readily inhale those toxic compounds as their blowhole is at the surface of the water (Venn-Watson et al. 2015a). Once weathering of dilbit is complete, a larger proportion of crude is expected to sink and adhere to sediments as compared to other commonly transported conventional crudes due to bitumen’s high density and viscosity. Sinking dilbit can become incorporated in the bedload of coastal environments with the potential to be remobilized during a storm (National Academies of Sciences, Engineering, and Medicine 2016) or remobilized by benthic feeders when foraging (Esler et al. 2018).

Research has focused on highly publicized oil spill disasters (Philibert et al. 2016), termed ‘hyper-spills’, such as the 1989 *Exxon Valdez* oil spill (EVOS) and the 2010 *Deepwater Horizon* (DWH) oil spill (Murphy et al. 2016). Both of these spills dramatically impacted marine mammals: the EVOS resulted in mortality of several thousand sea otters (*Enhydra lutris*) (Ballachey et al. 1994; Esler et al. 2018) and significantly impacted one pod and one transient population of killer whales (*Orcinus orca*) (Matkin et al. 2008). Furthermore, recovery of sea otter abundance required over 20 years, and both the affected killer whale pod and transient population have yet to recover to pre-spill numbers (Esler et al. 2018). Following the DWH oil spill in the Gulf of Mexico at least 160 marine mammals died (U.S. Fish and Wildlife Service 2011) and bottlenose dolphin (*Tursiops truncates*) reproductive rates were significantly decreased in a heavily contaminated bay for nearly four years afterward (Lane et al. 2015). The DWH spill was also linked to prolonging an unusual cetacean mortality event in the northern Gulf of Mexico that had begun prior to the spill (Venn-Watson et al. 2015a).

Within Canada, increased oil sands development in Northern Alberta and transport of dilbit (Philibert et al. 2016) by tanker on the coastal waters of British Columbia pose a risk of dilbit exposure to marine wildlife. Fauna at risk of exposure to marine petroleum spillage include the endangered southern resident killer whale population that resides year-round in British Columbia’s coastal waters (Ford et al. 2010, 2017) particularly because shipping traffic transects this population’s critical habitat (Lacy et al. 2015). Additionally humpback whales (*Megaptera novaeangliae*) are found along British Columbia’s coast from spring to fall (Dalla Rosa et al. 2012). In total, twenty-four marine mammal species (Harvey et al. 2017) are found in these coastal waters, including the commonly observed harbor porpoise (*Phocoena phocoena*), Dall’s porpoise (*Phocoenoides dalli*), Pacific white-sided dolphin (*Lagenorhynchus obliquidens*), minke whale (*Balaenoptera acutorostrata*), fin whale (*Balaenoptera physalus*), sea otter, northern elephant seal (*Mirounga angustirostris*), Steller sea lion (*Eumetopias jubatus*), and harbor seal (*Phoca vitulina*) (Williams et al. 2011a).

By reviewing the impacts of conventional crude oil to marine mammals and identifying common toxicity endpoints, this review will inform planned research on unconventional crude oil toxicity such as dilbit.

## Methods

A literature search was completed in Web of Science™. All years from 1900 to 2020 were included with no filters used. Search terms included a petroleum derivative, vertebrate class, and any words including toxic. Petroleum derivatives used in the search were “petroleum” OR “fuel” OR “hydrocarbon” OR “oil spill” OR “bitumen” OR “crude oil.” Vertebrate search terms included “mammal.” Search terms by topic were specifically “((petroleum OR fuel OR hydrocarbon OR “oil spill” OR bitumen OR “crude oil”) AND (mammal) AND (*toxic*))”. Resulting retrieved paper titles were categorized based on relevance. Few (24) relevant marine mammal papers were found through the Web of Science™ search, consequently select references within retrieved papers were also included in this review. As there were numerous references to two books *Marine Mammals and the Exxon Valdez* (Loughlin 1994) and *Sea Mammals and Oil; Confronting the Risks* (Geraci and St. Aubin 1990), relevant chapters from each book were included. Lastly, information from a Canadian Department of Fisheries and Oceans report (Dupuis and Ucan-Marin, 2015) and book chapter (Frasier et al. 2020) were added. In this way, we have tried to represent oil spill research on marine mammals as accurately and objectively as possible. Therefore, this review is based on information from 68 research papers or book chapters related to conventional petroleum impacts on marine mammals. Impacts of toxicity are summarized in Table 1.

**Table 1:** Percentage of papers or book chapters retrieved from the literature as categorized by type of petroleum or petroleum-related PAH impact

Impact	Percentage (%)
Acute mortality	20.8
Impacts associated with fouling of the pelage	13.9
Alteration of behaviour	12.5
Modulation of the HPA axis	6.9
Reduction of reproductive success	6.9
Chronic mortality	6.9
Hematological injury	5.6
Genotoxicity	5.6
Lung disease	5.6
Immune function	4.2
Eye irritation	4.2
Neurotoxicity	2.8
Liver lesions	2.8
Long-term population decline	2.8
Change in organ mass	1.4

### Risk of petroleum toxicity to marine mammals

Marine mammals are at risk of contact to petroleum through all routes of exposure (Neff 1990). For example, following the DWH oil spill, dolphins were observed swimming in oil contaminated waters. Consequently, the routes of exposure to petroleum for those dolphins included direct contact to oil both at the surface of the water and within the water column, inhalation of PAH volatiles at the air-water interface, incidental ingestion of contaminated water and sediment when foraging, and ingestion of contaminated prey (Schwacke et al. 2014). Inhalation of the toxic aromatic hydrocarbon vapours (BTEX), most concentrated above oil slicks, can readily be inhaled by those marine mammals with blowholes (Venn-Watson et al. 2015a). In particular, for those species that lack air-filtering cilia and nasal turbinates, such as dolphins, exposure to PAH volatiles upon inhalation would likely be exacerbated (Venn-Watson et al. 2015a). Inhalation of PAH volatiles caused brain lesions and resulted in the deaths of numerous harbor seals (Peterson 2001), as well as eye irritation and lacrimation in ringed (*Phoca hispida*) and grey seals (*Halichoerus grypus*) (Geraci and Smith 1976; Hall et al. 1996). For species with fur, such as fur seals, sea otters, and polar bears (*Ursus maritimus*) oiling of the pelage will decrease thermoregulation and result in hypothermia and death (Neff 1990). Additionally, oiling of the dermis of pinnipeds can reduce locomotion and in severe cases can cause drowning when the flippers are adhered to the body (Davis and Anderson 1976). Oil that has been washed ashore specifically in haul-out and nursery sites can foul pinnipeds and will eventually return to subtidal sediments whereby it may be ingested by grey whales (*Eschrichtius robustus*), walruses (*Odobenus rosmarus*) and seals that prey on benthic fauna (Neff 1990).

When oil compounds and hydrocarbons are absorbed into the circulation of marine mammals they attack the liver, nervous system, and blood-forming tissues (Geraci and St. Aubin 1990). However, marine mammals and their surrogate test species have the ability to metabolize petroleum hydrocarbons through the mixed function oxidase system (MFO) as quantified in the harbor seal, bottlenose dolphin, white-sided dolphin, harbor porpoise, minke whale, pinnipeds, and American mink (*Mustela vison*) (Engelhardt 1982; Geraci and St. Aubin 1982; Goksoyr et al. 1986; Watanabe et al. 1989; Frost et al. 1994b; Schwartz et al. 2004a; Lee and Anderson 2005). This metabolism has also been measured indirectly through elevated ethoxyresorufin-o-deethylase (EROD) activity in common minke whale liver microsomes incubated in the PAH phenanthrene (Goksoyr et al. 1986). EROD activity has also been measured in polar bears (Letcher et al. 1996; Mckinney et al. 2011), ringed seals (Mckinney et al. 2011), and beluga whales (*Delphinapterus leucas*) (Mckinney et al. 2011). The aryl hydrocarbon receptor, a common inducer of the transcription of MFO enzymes was likewise identified in beluga whales (Hahn et al. 1992).

However, the extent to which marine mammals can metabolize and eliminate hydrocarbons is unclear. Cetaceans as a group do not have sweat glands, sebaceous glands, or gills; consequently the routes cetaceans use to eliminate petroleum hydrocarbons may be limited and lipophilic contaminants such as PAHs may accumulate in blubber (Engelhardt 1983; Marsili et al. 2001). Additionally, marine mammals that have ingested hydrocarbons and then undergo a deep dive experience increased exposure to petroleum hydrocarbons. During deep dives hydrocarbons bypass the liver and its detoxifying activity, instead directly entering the brain and other tissues, which may result in death (Geraci et al. 1989). Moreover, breath holds allow for prolonged contact and exchange between inhaled PAH vapors and blood (Irving et al. 1941; Ridgway et al. 1969).

The specific habitats in which marine mammals live also have the potential to increase risk of exposure to oil. In North America over half of pinniped species reproduce on pack ice which can concentrate oil between ice floes or accumualte in flotsam (LeFèvre 1986) whereby it could be ingested (McLaren 1990). Oil may also persist at ice edges, leads, and breathing holes frequently visited by polar bears, narwhals (*Monodon monoceros*), belugas, ringed seals, and walruses (Neff 1990). Toxic volatiles from oil will evaporate slower in low Arctic temperatures, enhancing the toxicity of particularly light crudes (Ottway 1971). Moreover, oil can concentrate in bays and estuaries along the coastline frequently used by pinnipeds and cetaceans (McLaren 1990). Likewise diet influences exposure to oil hydrocarbons. Because bivalves such as molluscs are limited in their detoxifying capacity, they bioconcentrate hydrocarbons, frequently consumed by sea otters and walruses (Geraci and St. Aubin 1990). Similarly plankton, commonly consumed by baleen whales, can engulf oil droplets for up to ten days (Geraci and St. Aubin 1990).

Sediments may harbour lingering petroleum following an oil spill, increasing petroleum exposure to fauna that commonly forage there (Esler et al. 2018). For example, ingestion of PAH-contaminated sediment was proposed as a possible cause of digestive tract cancers found in an isolated population of beluga whales of the St. Lawrence Estuary (Lair et al. 2016). An estimated14% of 222 beluga carcasses from the St. Lawrence Estuary in Quebec, Canada necropsied from 1983–2012 had malignant neoplasms in their digestive tracts, the highest occurence of cancer reported for any population of cetaceans (Lair et al. 2016). Sediments in the Saguenay River contained high amounts of PAHs specifically during 1956–1976 due to waste contamination from nearby aluminum smelters. Because the specific foraging strategy employed by belugas involves ingestion of sediment when suction-feeding on benthic prey, it is likely these individuals directly ingested the PAHs bound to sediment (Lair et al. 2016). Digestive tract neoplasms occurred possibly due to chronic exposure to those highly carcinogenous PAHs (Lair et al. 2016).

Most knowledge of marine mammal oil toxicity data comes from the aftermath of oil spills as marine mammals are protected under legislation such as the Marine Mammal Protection Act (MMPA) and the Endangered Species Act (Takeshita et al. 2017). Laboratory toxicity studies on marine mammals in the 1970’s and 1980’s included tests species such as sea otters (Costa and Kooyman 1982), ringed seals (Geraci and Smith 1976), bottlenose dolphins (Geraci and St. Aubin 1982, 1985; Engelhardt 1983; Geraci et al. 1983; Smith et al. 1983; St. Aubin et al. 1985; Harvey and Dahlheim 1994), and a rare study involving three polar bears (Øritsland et al. 1981). More recently, however, petroleum toxicity studies are limited to use of surrogates such as mink in place of sea otters (Bickham et al. 1998; Mazet et al. 2001; Schwartz et al. 2004a, 2004b) and in vitro studies (Carvan et al. 1995; Reichert et al. 1999; Frouin et al. 2010; Wise et al. 2014; White et al. 2017; Wise et al. 2018a). The following sections review common impacts of petroleum exposure to marine mammals from both laboratory studies and oil spills.

### Hematological injury, immune function, and changes in organ weight

Exposure to crude oil can induce hematological injury and modulate both immune function and organ weight. For example, American mink exposed to 500 ppm bunker C fuel oil mixed in their feed over 113–118 days experienced changes in hematology and organ weight (Schwartz et al. 2004a, 2004b). Chronic ingestion of fuel oil was associated with a decrease in erythrocyte count, hemoglobin, and hematocrit, and an increase in mean corpuscular volume. Liver and adrenal weight increased (adrenal hypertrophy), mesenteric lymph node weight decreased, and hepatic cytochrome P4501A1 (CYP1A1) mRNA was elevated (Schwartz et al. 2004a). Additionally, chronic ingestion of fuel oil elicited multiple immune responses in American mink including elevation of various white blood cells, a significant increase in the absolute numbers of specific peripheral blood lymphocyte subsets, and increases in expression of both functionally significant cell surface proteins and mitogen-induced mononuclear cell proliferative responses (Schwartz et al. 2004b). Modulation of immune function also occurred in bottlenose dolphin lymphocytes following in vitro exposure to environmentally relevant concentrations of Louisiana sweet crude oil (1 liter/1 gram media/oil) causing both B- and T-cell proliferation of white blood cells to increase (White et al. 2017). Health assessments on bottlenose dolphins from DWH oil contaminated regions likewise revealed an increase in B and T lymphocyte proliferation as compared to conspecifics from unoiled regions (De Guise et al. 2017). Lastly, anemia was present in 4 of 32 individual dolphins exposed to DWH oil (Schwacke et al. 2014) and in polar bears after ingestion of Midale crude oil (Øritsland et al. 1981). Anemia was also reported in about half of the sea otter mortalities documented in rehabilitation centers following the EVOS (Rebar et al. 1995).

### Genotoxicity

Crude oil or its components have been reported to modify DNA in sea otters and their surrogate test species, American mink. Genome size increased in kidney samples from mink kits exposed to crude oil through diet and their mother’s milk for a duration of about four months (Bickham et al. 1998). A subsequent dosing study with either crude oil or bunker C fuel oil applied through the diet or externally to yearling female mink resulted in clastogenetic damage in spleen tissues (Bickham et al. 1998). Consequently, petroleum exposure in mink can cause somatic chromosomal damage and alteration of genome size (Bickham et al. 1998). Clastogenetic damage can also result from petroleum exposure in the field. Nearly two years following the EVOS, 30% of blood samples taken from sea otters living in petroleum contaminated areas of Prince William Sound revealed clastogenetic damage (Bickham et al. 1998). Additionally, necropsies of beluga whales native to the highly polluted St. Lawrence Estuary revealed benzo[a]pyrene adducts were present in liver and brain tissues in 10 of 11 individuals (Martineau et al. 1994).

In vitro studies with PAHs such as benzo(a)pyrene also report DNA damage. In bottlenose dolphin kidney cell lines, benzo[a]pyrene CYP1A1-mediated metabolites formed DNA adducts and inhibited mitosis in a dose dependant manner (Carvan et al. 1995). Genotoxic effects were also detected in a harbor seal cell line exposed to benzo[a]pyrene (Frouin et al. 2010). Lastly, DNA adducts were detected in hepatic tissue from harbor seal carcasses obtained from petroleum contaminated EVOS sites via the 32-P-postlabeling technique (Reichert et al. 1999). PAHs from petroleum were considered the cause of DNA damage due to the chromatographic profiles of the adducts (Reichert et al. 1999).

### Eye Irritation

Eye irritation is common in petroleum exposed seals. Following the *Braer* oil spill in 1993, upon inhalation of volatiles, grey seals had redness in the whites of the eyes, and eye infections (Hall et al. 1996). Additionally a twenty-four hour exposure to a 1 cm thick slick of Norman Wells crude oil in ringed seals resulted in temporary eye irritation including lacrimation, reddening and inflammation of the conjunctiva, and squinting (Geraci and Smith 1976). Necropsies of oiled harbor seals revealed higher incidence of conjunctivitis and skin irritation along with liver lesions in oiled seals as compared to those that were unexposed (Spraker et al. 1994).

### Neurotoxicity

Brain lesions, stress, disorientation, and acute mortality of at least 302 harbor seals following the EVOS were attributed to inhalation of short-chain petroleum volatiles (Peterson 2001). In the spring and summer of 1989, harbor seals were exposed to high concentrations of volatile petroleum hydrocarbons (up to 9 ppm) over oil slicks in Prince William Sound (Frost et al. 1994b). Elevation of the aliphatic hydrocarbon phytane (>1000 ppb) was found in the brains of seals from contaminated sites following the spill in 1989, but by 1990 levels of PAHs in the brain had decreased (Frost et al. 1994b). Four types of brain lesions, intramyelinic edema, axonal degeneration, neuronal swelling, and neuronal necrosis were present in oiled harbor seals as compared to unoiled seals (*P* < 0.01), characteristic of hydrocarbon toxicity. The brain lesions primarily occurred in the thalamus, likely explaining the disorientation and lethargy that was observed in harbor seals immediately following the spill and could have also contributed to difficulty swimming, feeding, or diving (Spraker et al. 1994).

### Lung disease

Cetaceans exposed to crude oil may have an increased incidence of lung disease. For example, in Louisiana, Mississippi, and Alabama, increased dolphin strandings were associated with significantly elevated PAHs in the water following the DWH oil spill (Venn-Watson et al. 2015a). Based on analysis of tissues from bottlenose dolphins that stranded in these states, stranded dolphins were more likely to have primary bacterial pneumonia (*P* = 0.003) as compared to dolphins from reference sites and primary bacterial pneumonia caused or contributed significantly to death in 70% of the strandings. Additionally, health assessments on bottlenose dolphins in the heavily oiled Barataria Bay, Louisiana, revealed pulmonary disease and lung lesions attributed to DWH oil; specifically, pulmonary abnormalities lingered (Schwacke et al. 2014) up to four years following the spill (Smith et al. 2017). Dolphins in Barataria Bay had a five-fold greater chance of having lung disease (moderate to severe) as compared to dolphins at a reference site. Lung disease was characterized by significant alveolar interstitial syndrome, lung masses, and pulmonary consolidation. 17% of those dolphins assessed in Barataria Bay were not expected to survive (Schwacke et al. 2014).

Exposure to crude oil may also cause lung disease in pinnipeds. Based on necropsy data of oiled harbor seals, inhalation of vapours from the EVOS may have casued interstitial pulmonary emphysema, leading to mortality of those seals that were even lightly oiled (Lipscomb et al. 1994). Additionally, of the oiled otters that died both in rehabilitation and in oil-contaminated sites of Prince William Sound, 41% and 66% had interstitial pulmonary emphysema. In contrast, only 21% of unoiled carcasses from the wild had interstitial pulmonary edema. Pulmonary emphysema occurred along with other abnormalities such as gastric erosions and hepatic lipidosis (Lipscomb et al. 1994).

### Impacts on the endocrine system and reproductive success

Petroleum can affect the hypothalamic-pituitary-adrenal (HPA) axis, specifically the adrenal gland, possibly because this gland can be a significant site for metabolism of PAHs (Venn-Watson et al. 2015a). Based on analysis of tissues from bottlenose dolphins that stranded in Louisiana, Mississippi, and Alabama following the DWH oil spill, stranded dolphins were more likely to have thin adrenal cortices (*P* = 0.003) as compared to dolphins from reference sites and petroleum exposure likely resulted in chronic adrenal insufficiency, increasing susceptibility to adrenal crises (Venn-Watson et al. 2015a). Additionally, health assessments on bottlenose dolphins in the heavily oiled Barataria Bay, Louisiana, revealed compromised stress response and adrenal lesions including hypoadrenocorticism; specifically, impaired stress response lingered (Schwacke et al. 2014) up to four years following the spill (Smith et al. 2017). These impacts were attributed to chronic petroleum exposure because the dolphin population in Barataria Bay exhibits strong site fidelity (Wells et al. 2017). In other studies, exposure to petroleum caused adrenal hypertrophy in mink (Schwartz et al. 2004a) and adrenal insufficiency was named as a possible cause of death in ringed seals following a twenty-four hour immersion in a one centimeter thick slick of Norman Wells crude oil (Geraci and Smith 1976).

Chronic exposure to crude oil may also impact the hypothalamic-pituitary-gonadal (HPG) axis. For example, a chronic study on mink (*Mustela vison*) used as surrogates for protected sea otters linked petroleum exposure to significantly reduced reproductive success (Mazet et al. 2001). Mink were exposed to either 0.065 g/kg body weight/day Alaskan North Slope crude or bunker C fuel oil and exposures began 60 days prior to breeding continuing until kits were weaned (Mazet et al. 2001). Result highlights include fewer births in the fuel oil dietary group (*P* < 0.05), a decreased average number of liveborn kits in dietary exposed groups as compared to controls (*P* < 0.05), and poor survival to weaning in dietary exposed groups (Mazet et al. 2001). The reproductive success of kits whose mothers were exposed to dietary fuel oil was also significantly reduced (*P* < 0.05) even though the kits themselves were exposed to fuel oil only through nursing or when in utero (Mazet et al. 2001). These results indicate that if sea otters in the wild were chronically exposed to petroleum through their diet, they may experience reduced reproductive success (Mazet et al. 2001).

Bottlenose dolphins from Barataria Bay, Louisiana, similarly had decreased reproductive success following the DWH oil spill. After monitoring 10 pregnant dolphins in a heavily oiled area for 47 months 20% of mothers in the heavily oiled bay produced viable calves, a significant drop from the reproductive success rate of 83% in a reference population (Lane et al. 2015). Moreover, the Barataria bay dolphin population had an increased annual mortality rate by up to 9%, above baseline rates. Those dolphins sustained reduced reproduction and survival rates following the spill indicating the effects of the oil spill were long-lasting; exposure to oil hydrocarbons was associated with poor maternal health which resulted in perinatal losses (Lane et al. 2015). Bottlenose dolphins from another DWH oil contaminated region of the Gulf of Mexico, Misssissippi Sound, had a similarly low reproductive success rate (19.4%) following the spill (Kellar et al. 2017).

Lastly, following large oil spills fewer offspring were produced by harp seals (*Pagophilus groenlandicus*) (Frost et al. 1994a) and sea otters (Tuomi and Williams 1995) while unusually high abortion rates and premature births were thought to occur in harbor seals (Spraker et al. 1994).

### Fouling of pelage: metabolic and clinical abnormalities

Petroleum reduces the fur’s insulative properties by removing the natural oils that waterproof it. For example, fouling of one third of the fur can result in 50% greater heat loss in fur seals (Kooyman et al. 1977). The marine mammal possibly most adversely affected by pelage fouling is the sea otter (Geraci and Williams 1990; Rebar et al. 1995; Williams et al. 1995; Schwartz et al. 2004a). While otters will avoid oil when given a choice, once contaminated they will obsessively groom (Geraci and Williams 1990) for hours, displacing other activities such as feeding (Ralls and Siniff 1990). Because their high metabolisms cannot tolerate interruptions in feeding, the intensive grooming, compromised insulation of fur, and oral ingestion of oil compound metabolic stess, resulting in death. This also directly affect metabolic rate; for example, when 18% of the body surface of an otter was coated in oil and, after swimming for 30 minutes, metabolic rate increased by 40% (Costa and Kooyman 1982). Similarly, fouling of 20% pelage of otters was linked to a twofold increase in metabolic rate. Grooming, while intensive, spreads petroleum deeper into pelage, displacing other behaviours. Once otters coated with oil, time spent grooming increases from 35% to 61%, time spent swimming increased by 7%, and time spent resting decreased by 37% (Davis et al. 1988). In particular, because of the high density of their fur, otter pups are at greatest risk of mortality when oiled (Geraci and Williams 1990). Moreover, the life histories of otters predispose them to oil exposure; they are coastal, have stong site fidelity, eat bivales that concentrate oil hydrocarbons, spend much of their time at the surface of the ocean in kelp beds which commonly concentrate oil, and group together in rafts, allowing for simultaneous contamination (Ralls and Siniff 1990).

Following the EVOS, the common symptoms of rescued oiled sea otters included hypothermia, lethargy, hemorrhagic diarrhea, seizures, hypoglycemia, and anorexia as well as clinical chemistry abnormalities (Lipscomb et al. 1994). Clinical abnormalities associated with otter deaths in rehabilitation centers within the first 10 days of intake were most common in those otters that were heavily oiled (Rebar et al. 1995). Mortality was commonly preceded by shock and development of shock was dependant on the degree of oiling on pelage (Rebar et al. 1995).

The European otter (*Lutra lutra L*.) while not strictly marine, is a coastal mustelid and largely piscivorous (Pierce and Boyle 1991). After the *Sullom Voe* oil spill in Shetland, Scotland, 13 European otters were confirmed dead (Baker et al. 1981). Cause of death was haemorrhagic gastroenteropathy thought to be due to ingestion of oil during grooming of the pelage. It was estimated that the proportion of local mortalities was between 15–50% (Baker et al. 1981). The same was true for harp seals when 4000 gallons of Bunker C oil spilled into the Gulf of St. Lawrence (St. Aubin 1990a). Up to 15,000 harp seals were coated in Bunker C oil and this was linked to an increase in mortality rates, as return rates of tagged harp seals were 25% lower; however, other factors may have contributed, such as reduced availability of habitat for birthing pups (Sergeant 1991). In contrast, coating the pelage of nine whitecoat harp seal pups with petroleum had no toxicological effect. While young whitecoat harp seals were completely coated with Norman Wells crude oil (100% coverage) for two consecutive days surprisingly the study did not report any adverse effects including no changes in behaviour, core temperature, or pathology (Geraci and Smith 1976).

An experimental dermal exposure with three polar bears placed in an oil covered pool, specifically in a slick of Midale crude oil (Øritsland et al. 1981) for 15–50 min, resulted in abnormal metabolic rate, clinical abnormalities, and mortality. One day after oiling metabolic rate increased by 27–86% (Hurst et al. 1982). Initially intensive grooming occurred but subsided after five days. Grooming resulted in oral ingestion of oil and skin irritation. The ingested oil caused vomiting and diarrhea while absorbed hydrocarbons were excreted by bile and urine. Twenty-nine days after the exposure two of the three bears had died. Clinical abnormalities included peripheral hemolysis, erythropoietic dysfunction, renal abnormalities, anemia, blood chemistry abnormalities and decreased thyroid hormone levels. Necropsies indicated degeneration of the kidney tubules, low-grade liver lesions, depressed lymphocyte activity, and fungus containing ulcers. However, death might also have been related to the stressful experimental protocol imposed on the bears as it was due to oil toxicity: the three bears were given an inadequate diet, limited drinking water, and sustained infected surgical incisions and injection sites (St. Aubin 1990b). Even so, these metabolic and clinical abnormalities may occur in oil exposed polar bears in the wild if overlaid on other stressors like food reduction and extreme temperatures (St. Aubin 1990b).

While spills are rare within the polar bear’s habitat, and fouling of these bears has not been reported in the wild (St. Aubin 1990b), polar bears can detect and avoid surface slicks of crude oil (Øritsland et al. 1981). Polar bears spend much time on ice floes, which concentrate surface oil and, along with freezing temperatures, slow oil degeneration and elimination (Stirling 1990). Additionally, polar bears could be oiled when hunting for seals in leads and breathing holes, where they submerge their head (Stirling 1990). Lastly, ingestion of oil is possible as polar bears clean themselves while feeding on carcasses and cubs and females will groom one another (Stirling 1990), showing no aversion to the taste of petroleum (St. Aubin 1990b).

In contrast, for non-fur bearing marine mammals, such as the bottlenose dolphin, the epidermis is a very effective barrier to petroleum compounds. Even when oil was massaged into superficial wounds for 30 minutes, healing time was not hindered (Geraci and St. Aubin 1982, 1985).

### Behavioural impacts

Altered behaviour or lack thereof, following petroleum exposure is varied. In captivity for example, petroleum exposure elicited abnormal behaviour in ringed seals (Geraci and Smith 1976), and unusual tameness, lethargy, and disorientation in *Exxon Valdez* oil exposed harbor seals in the field (Lowry et al. 1994; Spraker et al. 1994). Abnormal behaviour also occurred in oil fouled sea otters which groomed themselves obsessively for hours (Geraci and Williams 1990; Ralls and Siniff 1990). In contrast, following the EVOS, approximately 474 harbor seals in various locations within Prince William Sound were oiled (81% of 585 seals) but made no attempt to change behaviour: they did not avoid oiled haulout sites or oily water, and continued to use oiled habitats, giving birth to and caring for pups which also became oiled through nursing (Lowry et al. 1994).

Based on laboratory study results, bottlenose dolphins do display avoidance behaviour after contacting petroleum oil slicks and can detect and avoid a variety of oils in the light and dark by using both vision and echolocation (Geraci et al. 1983; Smith et al. 1983; St. Aubin et al. 1985). In contrast, following the 1990 *Mega Borg* oil spill, bottlenose dolphins were observed to swim through oil sheens and slicks (Smultea and Würsig 1995). Furthermore, following the the EVOS, killer whales were seen to swim through surface oil (Matkin et al. 2008). Others observed swimming and behaving normally in oiled waters include fin and humpback whales (Geraci 1990) and sea lions (Calkins et al. 1994). Aerial surveys of cetaceans indicated individuals swam near surface oil but rarely in the slicks (Sorensen et al. 1984). Following the DWH oil spill 13 species of cetaceans were observed to swim through petroleum contaminated waters, causing petroleum to be adhered to the epidermis (Dias et al. 2017). These included Atlantic spotted dolphin (*Stenella frontalis*), bottlenose dolphin, Cuvier’s beaked whale (*Ziphius cavirostris*), pantropical spotted dolphin (*Stenella attenuata*), pygmy sperm whale (*Kogia breviceps*), Risso’s dolphin (*Grampus griseus*), rough-toothed dolphin (*Steno bredanensis*), sperm whale (*Physeter macrocephalus*), spinner dolphin (*Stenella longirostris*), striped dolphin (*Stenella coeruleoalba*), and clymene dolphin (*Stenella clymene*) (Dias et al. 2017).

Ingestion of crude oil may also modify activity and sleep-like behaviours. DWH marine mammal response teams reported lethargy in oil-exposed live stranded dolphins they encountered (Wilkin et al. 2017). In addition, oral ingestion of a single dose of 75 ml Norman Wells crude did not result in any adverse toxicological effects in ringed seals; however, sleep-like behaviour in dosed ringed seals was modified, specifically dosed seals were active for four hours longer than controls before sleeping (Geraci and Smith 1976).

### Potential effects on gut bacteria and feeding

Petroleum could impact the digestive processes of certain marine mammals that rely on symbiotic bacteria to break down cellulose and obtain nutrients. As manatees consume sea grasses, retained for a long period of time in the gut, they have unique gastric glands and gut flora and fauna specialized for hindgut fermentation. Ingested oil may interfere with these processess and increased uptake of hydrocarbons may occur due to the long retention time of food in the gut (St. Aubin and Lounsbury 1990). Ingested oil may eradicate digestive flora, resulting in an inability to obtain nutrients which may result in starvation (Wikelski et al. 2001, 2002; Romero and Wikelski 2002). Petroleum may also indirectly impact the feeding of large cetaceans such as the baleen whale. Heavy oil was found to foul baleen plates and caused obstruction of the flow of water between plates in laboratory studies (Geraci and St. Aubin 1982, 1985; St. Aubin et al. 1984).

### Mortality and long term population impacts

Mortality in the field is difficult to accurately ascertain. Estimates are often based on carcass recovery such as those reported following the EVOS and DWH oil spills. Carcasses found following the EVOS included: 1011 sea otters, 19 harbor seals, 12 Stellar sea lions, (St. Aubin and Geraci 1994) and 37 cetacean species including: 26 gray whales, 5 harbor porpoise, 2 minke whales, 1 fin whale, and 3 whales that could not be identified to species (Loughlin 1994). The EVOS carcass recovery search effort coincided with the grey whale northern migration which may have contributed to the large number of grey whale carcasses found in addition, cause of death for the 37 cetaceans recovered could not be definitively determined (Loughlin 1994). According to a 2011 report, around 160 marine mammal deaths were attributed to the DWH oil spill. 13 live and 157 dead mammals were collected; additionally of the live mammals only 5 were released (U.S. Fish and Wildlife Service 2011). Hundreds of dolphin strandings were also reported. A 2017 paper describing the DWH marine mammal response effort reported 13 live and 178 dead stranded cetaceans collected from April 2010 to May 2011: live stranded cetaceans included 10 bottlenose dolphins, 2 spinner dolphins, and 1 clyme dolphin (Wilkin et al. 2017). However, based on historical carcass-detection rates of cetaceans in the Gulf of Mexico, it was estimated that only 2% cetacean carcasses are recovered in the Gulf of Mexico, consequently, following the DWH oil spill cetacean mortalities were likely underestimated (Williams et al. 2011b). Conversely, frequency of carcass detection search efforts may result in an overestimate of spill-related mortalities. Increased search effort following the DWH oil spill for stranded bottlenose dolphins had the capacity to increase rates of documented strandings to abnormal numbers (Pitchford et al. 2018). Further, carcass recovery cannot be used to estimate lasting population level impacts as it fails to address loss of reproductive potential and chronic impacts that would reduce both fecundity and survival (Schwacke et al. 2017). One solution is to address chronic population level impacts through use of age, sex, and class-structured population modelling (Schwacke et al. 2017).

The DWH spill was attributed to prolonging the duration of a multi-year unusual cetacean mortality event that had begun in February 2010 in the northern Gulf of Mexico prior to the spill (Venn-Watson et al. 2015a). 87% of cetaceans affected by the unusual mortality event were bottlenose dolphins (Litz et al. 2014). Following the spill, from April 30 to November 2 there were 121 strandings, and 1060 strandings from November 3 to December 14 (NOAA 2014). Dolphin strandings including perinatal dolphins increased by 3.5 to 4 times the upper limit of baseline strandings in the oil-contaminated waters of Alabama, Louisiana, and Mississippi from 2010–2011 (Venn-Watson et al. 2015b). In 2011 increased incidence of perinatal dolphin strandings was attributed to exposure to DWH oil, colder than usual temperatures, and freshwater runoff (Carmichael et al. 2012). In the heavily oiled Barataria Bay estimated annual dolphin survival rate was lower than reference site averages, ranging from 0.80–0.85 (95% CI: 0.77–0.90) for three years following the DWH oil spill (survival at reference sites averaged 0.95); however, abundance increased from 1300 individuals to an estimated 3100 conspecifics (McDonald et al. 2017). Additionally, low annual survival for bottlenose dolphins in Mississippi Sound the year following the DWH spill averaged 0.73 (95% CI: 0.67–0.78) (Mullin et al. 2017). Unfortunately, baseline data for Barataria Bay bottlenose dolphin abundance was unknown and consequently could not be compared to post-spill data (McDonald et al. 2017).

One year following the EVOS, 13 individuals from a single killer whale pod died, of which the majority were reproductive females and juveniles. The mortality rate for this pod during 1989 and 1990 was 19.4% and 20.7% respectively, significantly higher than historical rates (Matkin et al. 1994). The EVOS also had chronic effects on this species (Matkin et al. 2008). Analysis of killer whale census data in Prince William Sound from 1984–2005 indicated one resident pod (AB pod) and one transient population (AT1 population) were adversely affected; only 18 months following the spill mortality rate increased to 33% and 41% respectively (Matkin et al. 2008), as compared to the normal pod mortality rate, which is about 2.5% annually. Neither the resident pod nor the transient population had recovered to their pre-spill numbers 20 years later (Matkin et al. 2008). The acute mortalities of the spill had long-lasting implications; the small AB pod lost a disproportionate number of adult and juvenile females, thereby slowing its reproductive rate (Esler et al. 2018). Additionally, the loss of females following the spill from the already threatened AT1 population contributes to its likelihood of eventual extinction (Esler et al. 2018). In contrast humpback whales near the vicinity of the EVOS appeared to not be affected; particularly abundance, calving rates, and mortality did not change following the spill (von Ziegesar et al. 1994). However, pre-spill humpback whale estimates were based on searches in a small geographic range while post spill estimates and abundance was likely higher due to both increased search effort and wider geographic coverage of surveys (von Ziegesar et al. 1994).

Harbor seals and sea otters sustained the highest mortality rates of any marine mammal (Loughlin et al. 1996) following the EVOS. Sea otter carcasses from contaminated areas contained much higher concentrations of aliphatic and aromatic hydrocarbons (>8 x) than otters from unoiled sites (Mulcahy and Ballachey 1994). Of the 364 oiled otters that were rescued 53% were rehabilitated and returned to the wild, 32% died at the centers, while the remaining were distributed to marine aquaria (Geraci and Williams 1990). Of those returned to the wild, 45 adult sea otters were surgically implanted with radio transmitters to track subsequent survival (Hofman 1994). Eight months later over 25% of those adults had died (Hofman 1994). Additionally, six months following the spill, of the at least 4500 otters in the affected area, 886 carcasses were found (Irons et al. 1988); this number increased to 994 when including carcasses in Prince William Sound and deaths in rehabilitation centers (Lipscomb et al. 1994).

Of the sea otter casualties attributed to the EVOS, many were maternal, fetal, or neonate (Tuomi and Williams 1995). While reproduction rates from late 1989 to summer 1991 did not significantly diminish, long term survival of otter pups was impacted (Ballachey et al. 1994). Three years following the spill, survival of pups was lower in oiled regions of Prince William Sound when compared to pups in unoiled regions (Monnett and Rotterman 1992; Ballachey et al. 1994; Mazet et al. 2001). Of the approximately 30,000 sea otters in the Gulf of Alaska and Prince William Sound, it was estimated 3500–5500 otters died following the EVOS, reducing the population by about 18% (Hofman 1994). Similarly, harbor seal pups were especially impacted following the EVOS. Of 19 harbor seal carcasses collected, 15 were oiled and 13 of these were pups (Spraker et al. 1994). Petroleum toxicity and related stress was thought to be associated with an unusually high rate of abortions and premature births, as well as deaths of both pups and adults in heavily oiled areas (Spraker et al. 1994). Additionally, based on mortality estimates from haulout sites, at least 302 harbor seals died (Frost et al. 1994a) and 26% fewer pups were produced at oiled sites in 1989 (Frost et al. 1994a). Sea otter abundance recovered to pre-spill estimates nearly 25 years following the EVOS (Esler et al. 2018). Delayed recovery for those sea otters was attributed to exposure to lingering petroleum; exposure was exacerbated by life history traits and species specific behaviours such as high site fidelity and foraging habits (Esler et al. 2018).

## Summary

**Fig. 1:**
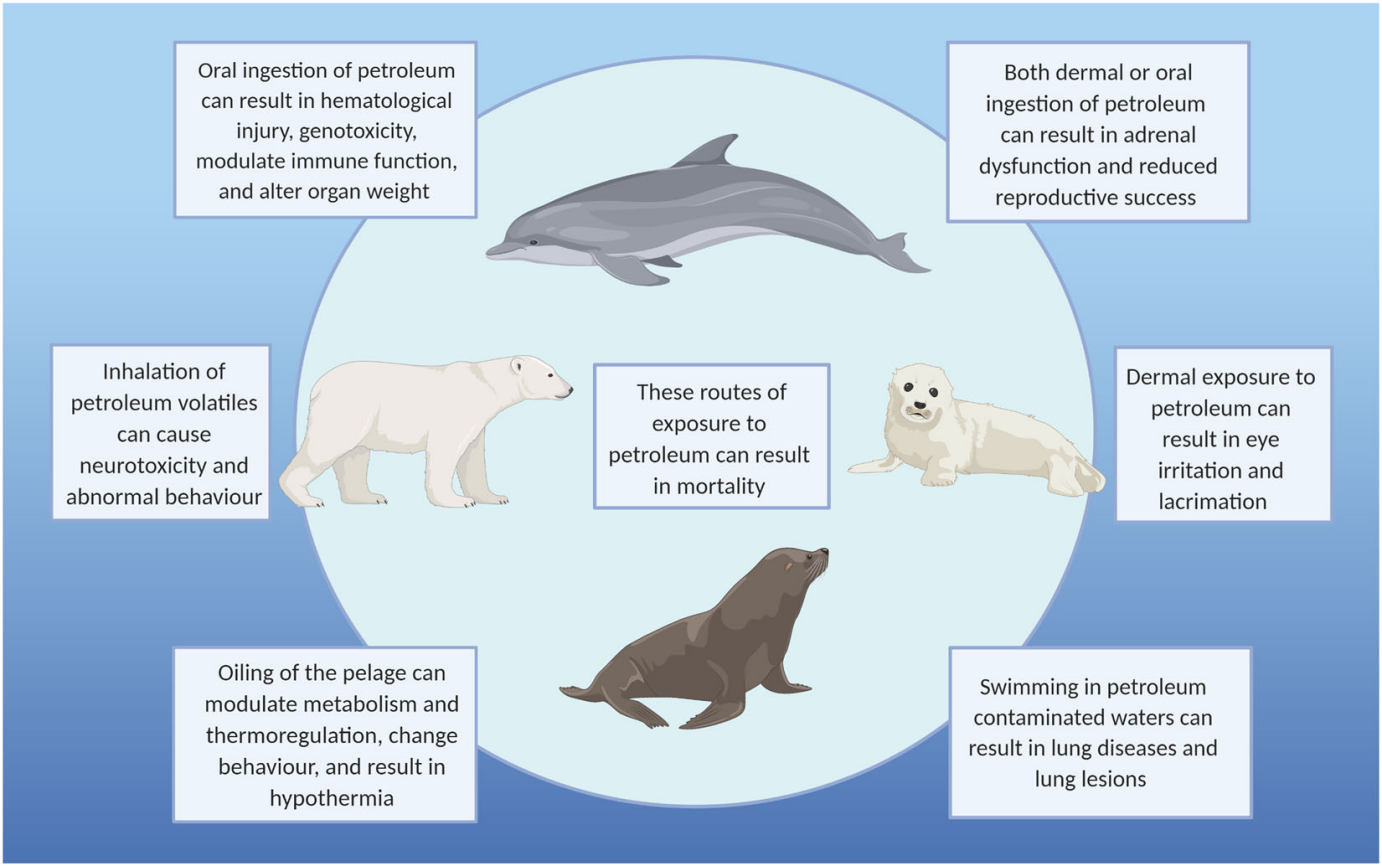
Common routes of petroleum exposure to marine mammals and consequent impacts of toxicity

Routes of exposure and toxicological impacts of petroleum to marine mammals are summarized in Fig. 1. On a taxonomic basis:

1. Effects on pinnipeds as a group include: behavioural abnormalities, eye irritation, liver and brain lesions, neurotoxicity, pulmonary emphysema, DNA damage, haemorrhagic gastroenteropathy, decreased reproductive success and mortality (Geraci and Smith 1976; Frost et al. 1994a; Lipscomb et al. 1994; Spraker et al. 1994; Hall et al. 1996; Loughlin et al. 1996; Reichert et al. 1999; Peterson 2001).
2. Cetaceans experience immune responses (De Guise et al. 2017; White et al. 2017) and DNA damage (Carvan et al. 1995). Of the cetaceans, petroleum exposure in bottlenose dolphins was linked to lung diseases, bacterial pneumonia, adrenal dysfunction, impaired stress response, lethargy, reduced reproductive success, and mortality (Schwacke et al. 2014; Lane et al. 2015; Venn-Watson et al. 2015a; Kellar et al. 2017; Smith et al. 2017; Wilkin et al. 2017).
3. Marine fissipeds such as sea otters succomb to hypothermia upon fouling of fur due to compromised insulation and metabolic stress, commonly resulting in death (Costa and Kooyman 1982; Geraci and Williams 1990; Ralls and Siniff 1990; Hofman 1994; Rebar et al. 1995).
4. Little toxicity data exists for polar bears (Øritsland et al. 1981) and no data exist for walruses and sirenians.While manatee habitat overlapped with the DWH oil footprint, none were observed while the spill was ongoing (Beyer et al. 2016); however, manatee populations in Florida are likely exposed regularly to hydrocarbons (Engelhardt 1983).

## Recommendations

1. Improved baseline data on PAH and metals exposure
  Measurement of PAHs, CYP upregulation, and metal concentrations in marine mammals through noninvasive methods such as use of biopsy darts would be useful for ongoing, long term monitoring of populations and contribute greatly to pre-spill data, especially for those marine mammals that share their coastal habitat with petroleum exploration and transport. Noninvasive methods would likewise assist in the monitoring of marine mammal species whereby there is little to no petroleum toxicity data available such as the manatee. Background levels of PAHs have been quantified in cetacean subcutaneous blubber using deploy of biopsy darts to free living cetaceans (Marsili et al. 2001) and CYP upregulation has been quantified in seven species of free living cetaceans through use of skin biopsy (Fossi et al. 2014). In addition, after the DWH spill, skin biopsies were collected from three cetacean species in the Gulf of Mexico to monitor metal concentrations in the skin, specifically those that are found in DWH petroleum (Wise et al. 2018b).
2. Improved pre- and post-spill data on marine mammal populations
  Although this may be challenging in some jurisdictions, baseline data regarding species abundance and demography is necessary to effectively assess the negative impacts of an oil spill to marine fauna and when insufficient, as in the case of both the EVOS and DWH oil spills, post-spill population assessment results are obfuscated (Bjorndal et al. 2011). For example, except for the ample baseline data for the bottlenose dolphins near Sarasota Bay, Florida (Wells 2014), data on marine mammal presence in the Gulf of Mexico was scarce prior to the 2010 DWH oil spill and abundance estimates were lacking, resulting in limited assessments of post-spill impacts to marine mammals (Frasier et al. 2020). A comparison of pre-and post-spill data is needed to effectively quantify the toxicological effects of petroleum (Frasier et al. 2020).
  Passive acoustic sensors represent an additional noninvasive tool for biomonitoring, specifically for those marine mammal species that use echolocation. With the ability to pick up individual echolocation clicking and pulses, passive acoustic sensors deployed during the DWH oil spill captured individual and species specific cetacean activity, part of a long term study (GOM High-frequency Acoustic Recording Package program) (Hildebrand et al. 2015; Frasier et al. 2017, 2020). However, because the sensors were not deployed prior to the spill, only post-spill data was obtained. While the passive acoustic sensor data implicated declines in some species specific activity, data was obfuscated by a lack of background knowledge regarding the various migratory ranges, seasonal patterns, and long term activity of cetaceans in the Gulf of Mexico (Frasier et al. 2020). Research is consequently needed, specifically on the long term activity of cetaceans to disentangle any potential impact of catastrophic petroleum spillage from the natural variability of populations (Aderhold et al. 2018). This includes long term research at the ecosystem level. For example, the herring population crash in Prince William Sound following the EVOS and subsequent lack of recovery may still be influencing predators at upper trophic levels such as marine mammals. Conversely, recovering marine mammal populations in Prince William Sound that feed on herring may be exerting top-down pressure on the herring population (Aderhold et al. 2018).
3. Use of surrogate mammalian models for petroleum toxicity testing
  Many knowledge gaps related to petroleum toxicity in marine mammals still exist and the protected status of marine mammals limit investigation to in vitro studies and noninvasive techniques. In light of both the limitations of field studies and current toxicity data available, we recommend that researchers continue to use species such as American mink as surrogates for marine mammals in petroleum toxicity dosing studies. It is crucial these toxicity tests be standardized according to dosage, duration of exposure, and test species. Comprehensive conventional petroleum toxicity data on marine mammal surrogates is especially needed in light of increasing transportation of unconventional petroleum with unknown toxicity such as diluted bitumen, which poses new risk to aquatic mammals.
4. Need for empirical data on toxicity of unconventional petroleum to marine mammals

A dosing study of dilbit and two conventional crude oils in zebrafish *(Danio rerio)* embryos determined that dilbit toxicity is equal to or less than conventional crude (Philibert et al. 2016). Extrapolating from that fish study, dilbit would pose a risk to marine mammals if spilled into the marine environment. In the event of a major spill, for example into Vancouver harbour, from a major proposed pipeline expansion, models estimate a greater than 50% probability that the Southern resident killer whale population would decrease dramatically (Lacy et al. 2015). Additionally, for the sea otter, one of the most vulnerable marine mammal species to oiling, a major spill of crude oil such as dilbit could greatly impact remaining endemic populations of sea otters already experiencing population pressures in British Columbia (Harris et al. 2011). While few dilbit dosing studies are available for aquatic fauna, this review highlights the common endpoints observed in conventional crude oil spills and exposures to marine mammals. These common endpoints inform planned research on unconventional crude oils such as dilbit toxicity and can assist in quantification of marine mammal health following spillage into the marine environment.

## References

[CR1] Aderhold DGR, Lindeberg MR, Holderied K, Pegau WS (2018). Spatial and temporal ecological variability in the northern Gulf of Alaska: what have we learned since the Exxon Valdez oil spill?. Deep Res Part II Top Stud Oceanogr.

[CR2] Anderson CM, LaBelle RP (1994). Comparative occurrence rates for offshore oil spills. Spill Sci Technol Bull.

[CR3] Baker JR, Jones AM, Jones TP, Watson HC (1981). Otter (Lutra lutra L.) mortality and marine oil pollution. Biol Conserv.

[CR4] Ballachey BE, Bodkin JL, DeGange AR, Loughlin TR (1994). An overview of sea otter studies. Marine mammals and the exxon valdez.

[CR6] Beyer J, Trannum HC, Bakke T (2016). Environmental effects of the deepwater horizon oil spill: a review. Mar Pollut Bull.

[CR7] Bickham JW, Mazet JA, Blake J (1998). Flow cytometric determination of genotoxic effects of exposure to petroleum in mink and sea otters. Ecotoxicology.

[CR8] Bjorndal KA, Bowen BW, Chaloupka M (2011). Better science needed for restoration in the Gulf of Mexico. Science (80-).

[CR9] Calkins DG, Becker E, Spraker TR, Loughlin TR, Loughlin TR (1994). Impacts on steller sea lions. Marine mammals and the exxon valdez.

[CR10] Carmichael RH, Graham WM, Aven A (2012). Were multiple stressors a ‘perfect storm’ for Northern Gulf of Mexico Bottlenose Dolphins (Tursiops truncatus) in 2011?. PLoS One.

[CR11] Carvan MJ, Flood LP, Campbell BD, Busbee DL (1995). Effects of Benzo(a)pyrene and Tetrachlorodibenzo(p)dioxin on fetal dolphin kidney cells: inhibition of proliferaton and initiation of DNA damage. Chemosphere.

[CR12] Costa DP, Kooyman GL (1982). Oxygen consumption, thermoregulation, and the effect of fur oiling and washing on the sea otter, *Enhydra lutris*. Can J Zool.

[CR13] Dalla Rosa L, Ford JKB, Trites AW (2012). Distribution and relative abundance of humpback whales in relation to environmental variables in coastal British Columbia and adjacent waters. Cont Shelf Res.

[CR14] Davis J, Anderson S (1976). Effects of oil pollution on breeding grey seals. Mar Pollut Bull.

[CR15] Davis RW, Williams TM, Thomas JA (1988). The effects of oil contamination and cleaning on sea otters (*Enhydra lutris*). II. Metabolism, thermoregulation, and behavior. Can J Zool.

[CR16] De Guise S, Levin M, Gebhard E (2017). Changes in immune functions in bottlenose dolphins in the northern Gulf of Mexico associated with the Deepwater Horizon oil spill. Endanger Species Res.

[CR17] Dew WA, Hontela A, Rood SB, Pyle GG (2015). Biological effects and toxicity of diluted bitumen and its constituents in freshwater systems. J Appl Toxicol.

[CR18] Dias LA, Litz J, Garrison L (2017). Exposure of cetaceans to petroleum products following the Deepwater Horizon oil spill in the Gulf of Mexico. Endanger Species Res.

[CR132] Dupuis A, Ucan-Marin F (2015). A literature review on the aquatic toxicology of petroleum oil: an overview of oil properties and effects to aquatic biota. DFO Can Sci Advis Sec Res Doc.

[CR19] Engelhardt FR (1982). Hydrocarbon metabolism and cortisol balance in oil-exposed ringed seals, *Phoca hispida*. Comp Biochem Physiol Part C Comp Pharmacol.

[CR20] Engelhardt RF (1983). Petroleum effects on marine mammals. Aquat Toxicol.

[CR21] Esler D, Ballachey BE, Matkin C (2018). Timelines and mechanisms of wildlife population recovery following the Exxon Valdez oil spill. Deep Res Part II Top Stud Oceanogr.

[CR22] Ford JKB, Pilkington JF, Reira A (2017). Habitats of special importance to resident killer whales (*Orcinus orca*) off the West Coast of Canada. DFO Can. Sci. Advis. Sec. Res. Doc.

[CR23] Ford JKB, Wright BM, Ellis GM, Candy JR (2010). Chinook salmon predation by resident killer whales: seasonal and regional selectivity, stock identity of prey, and consumption rates. DFO Can Sci Advis Sec Res Doc.

[CR24] Fossi MC, Panti C, Marsili L (2014). Could feeding habit and migratory behaviour be the causes of different toxicological hazard to cetaceans of Gulf of California (Mexico)?. Environ Sci Pollut Res.

[CR25] Frasier KE, Roch MA, Soldevilla MS (2017). Automated classification of dolphin echolocation click types from the Gulf of Mexico. PLoS Comput Biol.

[CR26] Frasier KE, Solsona-Berga A, Stokes L, Hildebrand JA, Murawski S (2020). Impacts of the deepwater horizon oil spill on marine mammals and sea turtles. Deep oil spills.

[CR27] Frost KJ, Lowry LF, Sinclair EH, Loughlin TR (1994). Impacts on Distribution, Abundance, and Productivity of Harbor Seals. Marine Mammals and the Exxon Valdez.

[CR28] Frost KJ, Manen CA, Wade TL, Loughlin TR (1994). Petroleum hydrocarbons in tissues of harbor seals from Prince William Sound and the Gulf of Alaska. Marine mammals and the exxon valdez.

[CR29] Frouin H, Fortier M, Fournier M (2010). Toxic effects of various pollutants in 11B7501 lymphoma B cell line from harbour seal (Phoca vitulina). Toxicology.

[CR30] Gauthier PT, Norwood WP, Prepas EE, Pyle GG (2014). Metal – PAH mixtures in the aquatic environment: a review of co-toxic mechanisms leading to more-than-additive outcomes. Aquat Toxicol.

[CR31] Geraci JR, Geraci JR, St. Aubin DJ (1990). Physiologic and toxic effects on cetaceans. Sea mammals and oil: confronting the risks.

[CR32] Geraci JR, Anderson DM, Tirnperi RJ (1989). Humpback whales (Megaptera novaeangliae) fatally poisoned by dinoflagellate toxin. Can J Fish Aquat Sci.

[CR33] Geraci JR, Smith TG (1976) Direct and indirect effects of oil on ringed seals (Phoca hispida) of the Beaufort Sea. J Fish Res Board Canada 33:1976–1984. 10.1139/f76-252

[CR34] Geraci JR, St. Aubin DJ (1982). Study of the effects of oil on Cetaceans..

[CR35] Geraci JR, St. Aubin DJ (1985). Expanded Studies of the Effects of Oil on Cetaceans..

[CR36] Geraci JR, St. Aubin DJ, Geraci JR, St. Aubin DJ (1990). Summary and conclusions. Sea mammals and oil: confronting the risks.

[CR37] Geraci JR, St. Aubin DJ, Reisman RJ (1983). Bottlenose dolphins, tursiops truncatus, can detect oil. Can J Fish Aquat Sci.

[CR38] Geraci JR, Williams TD, Geraci JR, St. Aubin DJ (1990). Physiologic and toxic effects on sea otters. Sea mammals and oil: confronting the risks.

[CR39] Goksoyr A, Solbakken JE, Tarlebo J, Klungsoyr J (1986). Initial characterization of the hepatic microsomal cytochrome P-450-system of the piked whale (Minke) Balaenoptera acutorostrata. Mar Environ Res.

[CR40] Hahn ME, Poland A, Glover E, Stegeman JJ (1992). The Ah receptor in marine animals: phylogenetic distribution and relationship to cytochrome P4501A inducibility. Mar Environ Res.

[CR41] Hall AJ, Watkins J, Hiby L (1996). The impact of the 1993 Braer oil spill on grey seals in Shetland. Sci Total Environ.

[CR42] Harris KA, Nichol LM, Ross PS (2011). Hydrocarbon concentrations and patterns in free-ranging sea otters (*Enhydra lutris*) from British Columbia, Canada. Environ Toxicol Chem.

[CR43] Harvey GKA, Nelson TA, Fox CH, Paquet PC (2017). Quantifying marine mammal hotspots in British Columbia, Canada. Ecosphere.

[CR44] Harvey JT, Dahlheim ME, Loughlin TD (1994). Cetaceans in oil. Marine mammals and the exxon valdez.

[CR45] Headley JV, McMartin DW (2004). A review of the occurrence and fate of naphthenic acids in aquatic environments. J Environ Sci Heal Part A.

[CR46] Hildebrand JA, Baumann-Pickering S, Frasier KE (2015). Passive acoustic monitoring of beaked whale densities in the Gulf of Mexico. Sci Rep.

[CR47] Hofman RJ, Loughlin TR (1994). Forward. Marine mammals and the exxon valdez.

[CR48] Hurst RJ, Øritsland NA, Watts PD (1982). Body mass, temperature and cost of walking in polar bears. Acta Physiol Scand.

[CR49] Irons DB, Nysewander DR, Trapp JL (1988). Prince william sound sea otter distribution in relation to population growth and habitat type.

[CR50] Irving L, Scholander PF, Grinnell SW (1941). The respiration of the porpoise, *Tursiops truncatus*. J Cell Comp Physiol.

[CR51] Kellar NM, Speakman TR, Smith CR (2017). Low reproductive success rates of common bottlenose dolphins *Tursiops truncatus* in the northern Gulf of Mexico following the Deepwater Horizon disaster (2010-2015). Endanger Species Res.

[CR52] Kennedy CJ (2015) Multiple effects of oil and its components in fish. In: Impacts of oil spill disasters on marine habitats and fisheries in North America, 1st edn. CRC Press pp 3–34

[CR54] Kooyman GL, Davis RW, Castellini MA, Wolfe DA (1977). Thermal conductance of immersed pinniped and sea otter pelts before and after oiling with Prudhoe Bay crude. Fate and effects of petroleum hydrocarbons in marine ecosystems and organisms.

[CR55] Lacy RC, Balcomb III KC, Brent LJN, et al. (2015) Report on Population Viability Analysis model investigations of threats to the Southern Resident Killer Whale population from Trans Mountain Expansion Project. Prepared for the National Energy Board (NEB) hearings reviewing Kinder Morgan’s proposed Trans Mountain Expansion Project. Raincoast Conservation Foundation. https://www.raincoast.org/wp-content/uploads/2015/05/RCF-SRKW-PVA-for-NEB-May-2015.pdf. Accessed September 2019

[CR56] Lair S, Measures LN, Martineau D (2016). Pathologic findings and trends in mortality in the Beluga (*Delphinapterus leucas*) Population of the St Lawrence Estuary, Quebec, Canada, from 1983 to 2012. Vet Pathol.

[CR57] Lane SM, Smith CR, Mitchell J (2015). Reproductive outcome and survival of common bottlenose dolphins sampled in Barataria Bay, Lousiana, USA following the Deepwater Horizon oil spill. Proc Biol Sci.

[CR58] Lee RF, Anderson JW (2005). Significance of cytochrome P450 system responses and levels of bile fluorescent aromatic compounds in marine wildlife following oil spills. Mar Pollut Bull.

[CR59] LeFèvre J (1986). Aspects of the biology of frontal systems. Adv Mar Biol.

[CR60] Letcher RJ, Norstrom RJ, Lin S (1996). Immunoquantitation and microsomal monooxygenase activities of hepatic cytochromes P4501A and P4502B and chlorinated hydrocarbon contaminant levels in polar bear (*Ursus maritimus*). Toxicol Appl Pharmacol.

[CR61] Lipscomb TP, Harris RK, Rebar AH, Loughlin TR (1994). Pathology of sea otters. Marine mammals and the exxon valdez.

[CR62] Litz JA, Baran MA, Bowen-stevens SR (2014). Review of historical unusual mortality events (UMEs) in the Gulf of Mexico (1990−2009): providing context for the multi-year northern Gulf of Mexico cetacean UME declared in 2010. Dis Aquat Organ.

[CR63] Loughlin TR, Loughlin TR (1994). Tissue hydrocarbon levels and the number of cetaceans found dead after the spill. Marine mammals and the exxon valdez.

[CR64] Loughlin TR, Ballachey BE, Wright BA (1996) Overview of studies to determine injury caused by the Exxon Valdez oil spill to marine mammals. In: American Fisheries Society Symposia vol. 18. pp 798–808. USGSPublications Warehouse. http://pubs.er.usgs.gov/publication/70007000

[CR65] Lowry LF, Frost KJ, Pitcher KW, Loughlin TR (1994). Observations of oiling of harbor seals in Prince William Sound. Marine mammals and the exxon valdez.

[CR66] Madison BN, Hodson PV, Langlois VS (2015). Diluted bitumen causes deformities and molecular responses indicative of oxidative stress in Japanese medaka embryos. Aquat Toxicol.

[CR67] Marsili L, Caruso A, Fossi MC (2001). Polycyclic aromatic hydrocarbons (PAHs) in subcutaneous biopsies of mediterranean cetaceans. Chemosphere.

[CR68] Martineau D, De Guise S, Fournier M (1994). Pathology and toxicology of beluga whales from the St. Lawrence Estuary, Quebec, Canada. Past, present and future. Sci Total Environ.

[CR69] Matkin CO, Ellis GM, Dahlheim ME, Zeh J, Loughlin TR (1994). Status of Killer Whales in Prince William Sound, 1985–1992. Marine Mammals and the Exxon Valdez.

[CR70] Matkin CO, Saulitis EL, Ellis GM (2008). Ongoing population-level impacts on killer whales *Orcinus orca* following the “Exxon Valdez” oil spill in Prince William Sound, Alaska. Mar Ecol Prog Ser.

[CR71] Mazet JAK, Gardner IA, Jessup DA, Lowenstine LJ (2001). Effects of petroleum on Mink applied as a model for reproductive success in sea otters. J Wildl Dis.

[CR72] McDonald TL, Hornsby FE, Speakman TR (2017). Survival, density, and abundance of common bottlenose dolphins in Barataria Bay (USA) following the Deepwater Horizon oil spill. Endanger Species Res.

[CR73] Mckinney MA, Dietz R, Sonne C (2011). Comparative hepatic microsomal biotransformation of selected PBDEs, including decabromodiphenyl ether, and decabromodiphenyl ethane flame retardants in arctic marine-feeding mammals. Environ Toxicol Chem.

[CR74] McLaren IA, Geraci JR, St. Aubin DJ (1990). Pinnipeds and oil: ecologic perspectives. Sea mammals and oil: confronting the risks.

[CR75] Monnett C, Rotterman LM (1992). Mortality and reproduction of female sea otters in Prince William Sound, Alaska.

[CR76] Mulcahy DM, Ballachey BE, Loughlin TR (1994). Hydrocarbon residues in sea otter tissues. Marine mammals and the exxon valdez.

[CR77] Mullin KD, McDonald T, Wells RS (2017). Density, abundance, survival, and ranging patterns of common bottlenose dolphins (*Tursiops truncatus*) in Mississippi Sound following the Deepwater Horizon oil spill. PLoS One.

[CR78] Murphy D, Gemmell B, Vaccari L (2016). An in-depth survey of the oil spill literature since 1968: Long term trends and changes since Deepwater Horizon. Mar Pollut Bull.

[CR79] National Academies of Sciences, Engineering, and Medicine (2016). Spills of diluted bitumen from pipelines: a comparative study of environmental fate, effects, and response.

[CR80] Neff JM (1979). Polycyclic aromatic hydrocarbons in the aquatic environment. Sources, fates and biological effects.

[CR81] Neff JM, Geraci JR, St. Aubin DJ (1990). Composition and fate of petroleum and spill-treating agents in the marine environment. Sea mammals and oil: confronting the risks.

[CR82] NOAA (2014) 2010-2014 Cetacean Unusual Mortality Event in Northern Gulf of Mexico. National Oceanic and Atmospheric Administration. Office of Protected Resources

[CR83] Øritsland NA, Engelhardt FR, Juck FA (1981). Effects of Crude Oil on Polar Bears.

[CR84] Ottway S (1971) The comparative toxicities of crude oils. In: The ecological effects of oil pollution on littoral communities. Inst. of Petroleum, pp 172–180

[CR85] Peterson CH (2001). The “Exxon Valdez” oil spill in Alaska: acute, indirect and chronic effects on the ecosystem. Adv Mar Biol.

[CR86] Philibert DA, Philibert CP, Lewis C, Tierney KB (2016) Comparison of diluted bitumen (Dilbit) and conventional crude oil toxicity to developing zebra fish. 10.1021/acs.est.6b00949 10.1021/acs.est.6b00949 27176092

[CR87] Pierce GJ, Boyle PR (1991). A review of methods for diet analysis in piscivorous marine mammals. Ocean Mar Biol Annu Rev.

[CR88] Pitchford JL, Garcia M, Pulis EE (2018). Gauging the influence of increased search effort on reporting rates of bottlenose dolphin (*Tursiops truncatus*) strandings following the deepwater horizon oil spill. PLoS One.

[CR89] Ralls K, Siniff DB, Ralls K, Siniff DB (1990) Sea otters and oil: ecologic perspectives. In: Geraci JR, St. Aubin DJ (eds) Sea mammals and oil: confronting the risks. Academic Press Inc, San Diego, pp 199–209

[CR90] Rebar AH, Lipscomb TP, Harris RK, Ballachey BE (1995). Clinical and clinical laboratory correlates in sea otters dying unexpectedly in rehabilitation centers following the exxon valdez oil spill. Vet Pathol.

[CR91] Reichert WL, French BL, Stein JE (1999). Exposure of marine mammals to genotoxic environmental contaminants: application of the 32P-postlabelling assay for measuring DNA-Xenobiotic adducts. Environ Monit Assess.

[CR92] Ridgway SH, Scronce BL, Kanwisher J (1969). Respiration and deep diving in the bottlenose porpoise. Science.

[CR93] Romero LM, Wikelski M (2002). Severe effects of low-Level oil contamination on wildlife predicted by the corticosterone-stress response: preliminary data and a research agenda. Spill Sci Technol Bull.

[CR94] Schwacke LH, Smith CR, Townsend FI (2014). Health of common bottlenose dolphins (*Tursiops truncatus*) in Barataria Bay, Louisiana, following the Deepwater Horizon oil spill. Environ Sci Technol.

[CR95] Schwacke LH, Thomas L, Wells RS (2017). Quantifying injury to common bottlenose dolphins from the Deepwater Horizon oil spill using an age-, sex- and class-structured population model. Endanger Species Res.

[CR96] Schwartz JA, Aldridge BM, Lasley BL (2004). Chronic fuel oil toxicity in American mink (*Mustela vison*): systemic and hematological effects of ingestion of a low-concentration of bunker C fuel oil. Toxicol Appl Pharmacol.

[CR97] Schwartz JA, Aldridge BM, Stott JL, Mohr FC (2004). Immunophenotypic and functional effects of bunker C fuel oil on the immune system of American mink (*Mustela vison*). Vet Immunol Immunopathol.

[CR98] Sergeant DE (1991). Harp seals, man and ice. Can Spec Publ Fish Aquat Sci.

[CR99] Smith CR, Rowles TK, Hart LB (2017). Slow recovery of Barataria Bay dolphin health following the Deepwater Horizon oil spill (2013-2014), with evidence of persistent lung disease and impaired stress response. Endanger Species Res.

[CR100] Smith TG, Geraci JR, St. Aubin DJ (1983). Reaction of bottlenose dolphins, tursiops truncatus, to a controlled oil spill. Can J Fish Aquat Sci.

[CR101] Smultea MA, Würsig B (1995). Behavioral reactions of bottlenose dolphins to the Mega Borg oil spill Gulf of Mexico 1990.. Aquat Mamm.

[CR102] Sorensen PW, Medved RJ, Hyman MAM, Winn HE (1984). Distribution and abundance of cetaceans in the vicinity of human activities along the continental shelf of the Northwestern Atlantic. Mar Environ Res.

[CR103] Spraker TR, Lowry LF, Frost KJ, Loughlin TR (1994). Gross necropsy and histopathological lesions found in harbor seals. Marine mammals and the exxon valdez.

[CR104] St. Aubin DJ, Geraci JR, St. Aubin DJ (1990). Physiologic and toxic effects on pinnipeds. Sea mammals and oil: confronting the risks.

[CR105] St. Aubin DJ, Geraci JR, St. Aubin DJ (1990). Physiologic and toxic effects on polar bears. Sea mammals and oil: confronting the risks.

[CR106] St. Aubin DJ, Geraci JR, Loughlin TR (1994). Summary and conclusions. Marine mammals and the exxon valdez.

[CR107] St. Aubin DJ, Geraci JR, Smith TG, Friesen TG (1985). How do bottlenose dolphins, tursiops truncatus, react to oil films under different light conditions?. Can J Fish Aquat Sci.

[CR108] St. Aubin DJ, Lounsbury V, Geraci JR, St. Aubin DJ (1990). Oil effects on manatees: evaluating the risks. Sea mammals and oil: confronting the risks.

[CR109] St. Aubin DJ, Stinson RH, Geraci JR (1984). Aspects of the structure and composition of baleen, and some effects of exposure to petroleum hydrocarbons. Can J Zool.

[CR110] Stirling I, Geraci JR, St. Aubin DJ (1990). Polar bears and oil: ecologic perspectives. Sea mammals and oil: confronting the risks.

[CR111] Takeshita R, Sullivan L, Smith C (2017). The Deepwater Horizon oil spill marine mammal injury assessment. Endanger Species Res.

[CR112] Tuomi PA, Williams TM, Williams TM, Davis RW (1995). Rehabilitation of pregnant sea otters and females with newborn pups. Emergency care and rehabilitation of oiled sea otters: a guide for oil spills involving fur-bearing marine mammals.

[CR113] U.S. Fish and Wildlife Service (2011) Deepwater Horizon Response Consolidated Fish and Wildlife Collection Report. USFWS and NOAA. https://www.fws.gov/home/dhoilspill/pdfs/ConsolidatedWildlifeTable042011.pdf. Accessed September 2020

[CR114] Venn-Watson S, Colegrove KM, Litz J (2015). Adrenal gland and lung lesions in Gulf of Mexico Common Bottlenose Dolphins (*Tursiops truncatus*) found dead following the deepwater horizon oil spill. PLoS One.

[CR115] Venn-Watson S, Garrison L, Litz J (2015). Demographic clusters identified within the Northern Gulf of Mexico Common Bottlenose Dolphin (*Tursiops truncatus*) Unusual Mortality Event: January 2010 – June 2013. PLoS One.

[CR116] von Ziegesar O, Miller E, Dahlheim ME, Loughlin TD (1994). Impacts on Humpback Whales in Prince William Sound. Marine mammals and the exxon valdez.

[CR117] Watanabe S, Shimada T, Nakamura S (1989). Specific profile of liver microsomal cytochrome P-450 in dolphin and whales. Mar Environ Res.

[CR118] Wells RS, Yamagiwa J, Karczmarski L (2014). Social structure and life history of bottlenose dolphins near sarasota bay, Florida: insights from four decades and five generations. Primates and cetaceans. primatology monographs.

[CR119] Wells RS, Schwacke LH, Rowles TK (2017). Ranging patterns of common bottlenose dolphins *Tursiops truncatus* in Barataria Bay, Louisiana, following the Deepwater Horizon oil spill. Endanger Species Res.

[CR120] White ND, Godard-Codding C, Webb SJ (2017). Immunotoxic effects of in vitro exposure of dolphin lymphocytes to Louisiana sweet crude oil and Corexit^TM^. J Appl Toxicol.

[CR121] Wikelski M, Romero LM, Snell HL (2001). Marine iguanas oiled in the Galápagos. Science.

[CR122] Wikelski M, Wong V, Chevalier B (2002). Marine iguanas die from trace oil pollution. Nature.

[CR123] Wilkin SM, Rowles TK, Stratton E (2017). Marine mammal response operations during the Deepwater Horizon oil spill. Endanger Species Res.

[CR124] Williams R, Ashe E, O’Hara PD (2011). Marine mammals and debris in coastal waters of British Columbia, Canada. Mar Pollut Bull.

[CR125] Williams R, Gero S, Bejder L (2011). Underestimating the damage: interpreting cetacean carcass recoveries in the context of the Deepwater Horizon/BP incident. Conserv Lett.

[CR126] Williams TM, McBain JF, Tuomi PA, Wilson RK, Williams TM, Davis RW (1995). Initial clinical evaluation, emergency treatments, and assessment of oil exposure. Emergency care and rehabilitation of oiled sea otters: a guide for oil spills involving fur-bearing marine mammals.

[CR127] Wise CF, Wise JTF, Wise SS (2014). Chemical dispersants used in the Gulf of Mexico oil crisis are cytotoxic and genotoxic to sperm whale skin cells. Aquat Toxicol.

[CR128] Wise CF, Wise JTF, Wise SS, Wise JP (2018). Chemically dispersed oil is cytotoxic and genotoxic to sperm whale skin cells. Comp Biochem Physiol Part - C Toxicol Pharmacol.

[CR129] Wise JP, Wise JTF, Wise CF (2018). A three year study of metal levels in skin biopsies of whales in the Gulf of Mexico after the Deepwater Horizon oil crisis. Comp Biochem Physiol Part - C Toxicol Pharmacol.

[CR130] Woods J, Kung J, Kingston D (2008). Canadian crudes: a comparative study of SARA fractions from a modified HPLC separation technique. Oil Gas Sci Technol.

[CR131] Ylitalo GM, Collier TK, Anulacion BF (2017). Determining oil and dispersant exposure in sea turtles from the northern Gulf of Mexico resulting from the Deepwater Horizon oil spill. Endanger Species Res.

